# Quality of life of people with human immunodeficiency virus and seroconcordant and serodiscordant partnerships

**DOI:** 10.1590/0034-7167-2024-0569

**Published:** 2025-10-03

**Authors:** Larissa Rodrigues Siqueira, Gilmara Holanda da Cunha, Marcos Venícios de Oliveira Lopes, Maria Elisa Curado Gomes, Ane Kelly Lima Ramalho, Maiara Bezerra Dantas, Vanessa Sousa dos Santos, Giovanna Soares Lins

**Affiliations:** I Universidade Federal do Ceará. Fortaleza, Ceará, Brazil

**Keywords:** HIV, Quality of Life, Sexual Partners, Nursing, Health Promotion, VIH, Calidad de Vida, Parejas Sexuales, Enfermería, Promoción de la Salud

## Abstract

**Objectives:**

to assess quality of life of people with HIV with seroconcordant and serodiscordant partnerships.

**Methods:**

a cross-sectional study, carried out in three outpatient clinics with 190 people with HIV (95 with seroconcordant and 95 serodiscordant partnerships), using characterization form and quality of life assessment instrument. Descriptive statistics and associations among variables were performed, considering p-value<0.05 as statistically significant.

**Results:**

the majority had a higher perception of quality of life, with no difference between groups. Females had lower scores in all domains. Variables associated with unsatisfactory quality of life were having children, living with more than three people, unemployment, income below the minimum wage, CD4+T lymphocytes below 350 cells/mm^3^, detectable viral load, not accepting diagnosis, stigma, time in the prison system and dissatisfaction with healthcare follow-up.

**Conclusions:**

perceived quality of life was higher for most seroconcordant and serodiscordant individuals, with no difference between groups.

## RESUMEN

**Objetivos:**

evaluar la calidad de vida de personas con VIH con parejas seroconcordantes y serodiscordantes.

**Métodos:**

estudio transversal, realizado en tres consultorios externos con 190 personas con VIH (95 con parejas seroconcordantes y 95 parejas serodiscordantes), utilizando un formulario de caracterización y un instrumento de evaluación de la calidad de vida. Se realizó estadística descriptiva y asociaciones entre variables, considerándose como estadísticamente significativo un valor p<0,05.

**Resultados:**

la mayoría tuvo una percepción superior de calidad de vida, sin diferencias entre los grupos. Las mujeres obtuvieron puntuaciones más bajas en todos los dominios. Las variables asociadas a calidad de vida insatisfactoria fueron tener hijos, vivir con más de tres personas, desempleo, ingresos menores a un salario mínimo, linfocitos TCD4+ menores a 350 células/mm3, carga viral detectable, no aceptar el diagnóstico, estigma, tiempo en el sistema penitenciario e insatisfacción con la atención en salud.

**Conclusiones:**

la percepción de calidad de vida fue superior para la mayoría de los individuos seroconcordantes y serodiscordantes, sin diferencias entre los grupos.

## INTRODUCTION

Diagnosis of human immunodeficiency virus (HIV) infection initially involves acceptance of serology, which can be associated with guilt, fear and disappointment^(1,2)^. Early diagnosis enables the initiation of antiretroviral therapy (ART), which provides viral suppression, reduced mortality and increased survival^(3-5)^. An undetectable viral load is untransmittable^(6)^, but cases of failure of sustained suppression may occur and the viral load may become detectable again^(7)^, which shows the importance of monitoring it^(8)^ and using combination prevention methods to reduce the incidence of HIV^(9)^.

Adequate adherence to ART improves quality of life (QoL) of people with HIV^(3,4)^. QoL is an individual’s perception of their position in life, cultural context and value system in relation to their goals, expectations, standards and concerns^(10)^. It can be measured using instruments, but there is one specific for people with HIV, which assesses QoL multidimensionally, through the physical, psychological, level of independence, social relationships, environment, spirituality/religion/beliefs domains^(10)^. QoL of people with HIV can be harmed by stigma in emotional and sexual relationships, fear of abandonment and rejection for disclosing diagnosis^(11-13)^. Sexuality influences QoL, as positive HIV serology can reduce emotional involvement, due to the risk of transmission to partners^(14)^. Thus, people with HIV need guidance about their sexual partnerships and the importance of an undetectable viral load in order to improve aspects of sexual health and the ability to disclose diagnosis^(15)^.

The influence of relationships on QoL of this population stands out, as having a partner can mean greater social support and better QoL^(16)^. It is also important to assess QoL of people with HIV with different types of sexual partners, whether they are seroconcordant or serodiscordant, as a study found that QoL was better in the group without the virus, which indicates that people with HIV may have an unsatisfactory QoL when compared to the general population^(12)^.

Considering the advances achieved with ART^(3,4)^ and its effects on QoL^(14,16)^, it is relevant to assess QoL of people with HIV with seroconcordant and serodiscordant partnerships. Although there are studies on the subject^(16-18)^ and that compare serodiscordant and seroconcordant partners^(12,19)^, updated research on QoL in different geographic regions is important, especially after the coronavirus-19 (COVID-19) pandemic, which restructured some healthcare services^(20)^. These analyses can detect the aspects of life most affected by the disease and help healthcare professionals and public policy makers to better target their actions^(21)^. Given the above, the hypothesis of this study is that people with HIV with a serodiscordant partnership have a more unsatisfactory QoL than seroconcordant partners.

## OBJECTIVES

To assess QoL of people with HIV with seroconcordant and serodiscordant sexual partners.

## METHODS

### Ethical aspects

The project was approved by the Research Ethics Committee *Universidade Federal do Ceará* and co-participating institutions. This study followed Resolution 466/2012 of the Brazilian National Health Council. All participants signed the Informed Consent Form (ICF).

### Study design, period, place, population and sample

This is a cross-sectional study that followed STrengthening the Reporting of OBservational studies in Epidemiology recommendations^(22)^. Data collection took place from January 2022 to December 2023. The study was carried out in three public infectious disease outpatient clinics in Fortaleza, Ceará, Brazil, which are references in the care of people with HIV in the Northeast region. The population consisted of people with HIV who had a steady or occasional partnership, seroconcordant or serodiscordant. To calculate sample size, we considered the variation in the score of QoL instrument^(10)^ between people with HIV with seroconcordant and serodiscordant partnerships, a 95% confidence level, 80% power, and a 7% difference in scores between the two groups, using the formula below to compare means:


$$\begin{array}{c} N=\left(\sigma_{1}^{2}+\sigma_{2}^{2}\right)\cdot {\left(Z_{a/2}+Z_{1-\beta }\right)}^{2}\\ {\left(\mu_{2}-\mu_{1}\right)}^{2} \end{array}$$

The sample consisted of 90 participants per group. Due to the asymmetry of values obtained on scales, Pitman’s correction was used, calculating 95 participants per group, with a total of 190 people with HIV. Sampling was non-probabilistic for convenience. Inclusion criteria comprised people with HIV of both sexes, over 18 years of age, sexually active, with a regular or occasional sexual partner, seroconcordant or serodiscordant. Exclusion criteria comprised pregnant women.

### Study protocol

People with HIV were invited to participate in the study while they were waiting for their biannual medical appointment. The study was explained to them, and those who agreed to participate signed the ICF. The interview was conducted in a private office and lasted an average of 30 minutes. Two instruments were used. The Sociodemographic, Clinical, Epidemiological and Vulnerability Characterization Form for People with HIV^(23)^ included the following variables: age; sex; education; marital status; occupational status; number of people in the household; monthly family income; time since diagnosis and ART; sexual orientation; living with a partner; partner’s serostatus; number of children; CD4+ T lymphocyte count; viral load; acceptance of diagnosis; experience of stigma; time in the prison system; and satisfaction with the healthcare service.

QoL assessment instrument, the World Health Organization Quality of Life Instrument - HIV/AIDS module (WHOQOL-HIV-Bref), was developed by researchers linked to the World Health Organization in Portuguese and validated in Brazil^(10)^. The original questionnaire has 120 questions, 29 facets and one facet on general QoL and general health perception, and six domains: I. Physical (pain, discomfort, energy, fatigue, sleep, rest); II. Psychological (positive and negative feelings, cognition, self-esteem, body image, appearance); III. Level of independence (mobility, activities of daily living, dependence on medication and treatments, suitability for work); IV. Social relationships (personal relationships, social support, sexual activity); V. Environment (physical safety, housing, finances, access to health and social assistance, ability to acquire information and learn new skills, leisure, physical environment, transportation); VI. Spirituality/religion/beliefs (forgiveness, guilt, concerns about the future, death and dying)^(10)^.

In this study, the abbreviated version of the instrument, the WHOQOL-HIV-Bref, was used, with 31 questions and six domains, and the facets on general QoL and general health perception^(10)^. Questions are scored on a 5-point Likert scale, where 1 indicates a low and negative perception, and 5 indicates a high and positive perceived QoL. The scores range from 4 to 20 points, reflecting the worst and best perceived QoL, respectively. QoL of people with HIV was stratified as unsatisfactory and satisfactory, according to question 1 of the WHOQOL-HIV-Bref^(10)^. The characterization of perceived QoL by domain scores occurred as follows: 4 - 9.9 (lower); 10 - 14.9 (intermediate); and 15 - 20 (higher)^(24,25)^. Lower scores indicate a lower perceived QoL^(26)^.

### Data analysis and statistics

Exploratory analysis consisted of descriptive statistical tests, absolute and relative frequencies, means, medians, and standard deviations. The Shapiro-Wilk normality test was used to verify the distribution of scores. Depending on the results of homogeneity of variance and adherence to normal distribution, in cases of non-normality, the Mann-Whitney test was applied to compare mean scores among variables consisting of two groups, the Kruskal-Wallis test for those with more than two groups, and the Dunn test for post-hoc comparisons. To associate the variables and scores of WHOQOL-HIV-Bref domains, the chi-square test, Fisher’s exact test or Fisher-Freeman-Halton test were applied, and the z test for post-hoc comparisons. The significance level was set at 5%, considering p<0.05 as statistically significant. The Statistical Package for the Social Sciences version 23.0 was used for analyses.

## RESULTS

Of the total sample of 190 people with HIV, the majority were male (138; 72.7%), with self-reported brown skin color (67.3%), married/stable union (54.2%), without children (59.4%), employed (55.2%), monthly income greater than one minimum wage (64.2%), heterosexual (51.6%), in the sexual exposure category (93.1%) and living with a partner (54.8%). The majority had a CD4+ T lymphocyte count greater than 350 cells/mm^3^ (84.8%), undetectable viral load (87.9%), accepted diagnosis (90%) and were satisfied with healthcare follow-up (93.1%). Some had been diagnosed (51.6%) and on ART (56.9%) for less than eight years, suffered stigma/prejudice (24.2%), had sexual relations for money/drugs (12.7%) and had been in the prison system (2.6%).

Age ranged from 21 to 65 years, with means and standard deviations for seroconcordant individuals of 39.95 ± 10.61 and for serodiscordant individuals of 35.82 ± 10.22. The number of years of schooling ranged from zero to 20, with medians and interquartile ranges of 10 and 6 for soconcordant individuals and 8 and 5 for serodiscordant individuals. Monthly income ranged from R$150.00 to R$20,000.00, with medians and interquartile ranges of R$1,818.00 and R$1,800.00 for soconcordant individuals and R$2,000.00 and R$2,400.00 for serodiscordant individuals.

The groups of people with HIV with seroconcordant and serodiscordant partners were similar in all WHOQOL-HIV-Bref domains, with data not adhering to normal distribution (p<0.05). The medians in the domains for seroconcordant individuals were 15.00 (I), 16.00 (II), 15.00 (III), 16.00 (IV), 14.50 (V) and 17.00 (VI). For serodiscordant individuals, these values were 15.00 (I), 16.00 (II), 14.00 (III), 16.00 (IV), 15.00 (V) and 17.00 (VI). Most people with HIV had a higher perceived QoL, with a similar analysis between those with seroconcordant and serodiscordant sexual partners, with no statistically significant difference being observed. In QoL classification by domain, there was no difference between seroconcordant and serodiscordant people with HIV, only a trend towards statistical significance in domain III (level of independence), in which serodiscordant people had a more intermediate perceived QoL compared to seroconcordant people (Table 1).

**Table 1:** Quality of life of people with HIV with seroconcordant and serodiscordant partners, average rank values by domain (N=190), Fortaleza, Ceará, Brazil, 2023

WHOQOL-HIV-Bref domains	Seroconcordant	Serodiscordant	X^2^	gl	U	*p* value
	n (%)	n (%)				
Average ranks						
Domain I	89.57	101.43			3949.50	0.136
Domain II	88.93	102.07			3888.00	0.098
Domain III	94.42	96.58			4410.00	0.785
Domain IV	88.18	102.82			3817.00	0.064
Domain V	88.07	102.93			3806.50	0.062
Domain VI	97.66	93.34			4307.50	0.586
Quality of life classification						
I - Physical						
Lower	9 (9.5)	6 (6.3)	1.0	2		0.587
Intermediate	34 (35.8)	31 (32.6)				
Higher	52 (54.7)	58 (61.1)				
II - Psychological						0.506^§^
Lower	5 (5.3)	3 (3.2)	-	-		
Intermediate	33 (34.7)	27 (28.4)				
Higher	57 (60.0)	65 (68.4)				
III - Level of independence						0.058^§^
Lower	7 (7.4)	2 (2.1)	-	-		
Intermediate	34 (35.8)	48 (50.5)				
Higher	54 (56.8)	45 (47.4)				
IV - Social relationships						0.455^§^
Lower	2 (2.1)	1 (1.1)	-	-		
Intermediate	34 (35.8)	27 (28.4)				
Higher	59 (62.1)	67 (70.5)				
V - Environment						0.271^§^
Lower	1 (1.1)	2 (2.1)	-	-		
Intermediate	49 (51.5)	38 (40.0)				
Higher	45 (47.4)	55 (57.9)				
VI - Spirituality/religion/beliefs						0.145^§^
Lower	5 (5.3)	1 (1.1)	-	-		
Intermediate	26 (27.3)	34 (35.7)				
Higher	64 (67.4)	60 (63.2)				

X^2^ - chi-square test; df - degrees of freedom; U - Mann-Whitney U test;

§ Fisher’s exact test.

As there was no difference between seroconcordant and serodiscordant groups, an analysis was performed with the total sample by WHOQOL-HIV-Bref domain. According to the scores, considering the 190 people with HIV, median, interquartile range and p-value were identified, respectively, of: domain I (15.00; 5.00; p<0.001); domain II (16.00; 4.00; p<0.001); domain III (15.00; 3.00; p<0.001); domain IV (16.00; 3.00; p<0.001); domain V (15.00; 2.60; p<0.002); domain VI (17.00; 5.00; p<0.001). The scores by domain indicated that the majority had a higher perceived QoL. In the analysis of general QoL, based on the first question of the instrument, only 21.6% of the sample had unsatisfactory QoL (median: 4.00).

In the analysis of the mean scores of sociodemographic variables associated with WHOQOL-HIV-Bref domains, females had lower scores in domains I (physical) (p=0.018), II (psychological) (p=0.017), III (level of independence) (p=0.021), IV (social relationships) (p=0.036), V (environment) (p=0.008) and VI (spirituality/religion/beliefs) (p=0.017). Those over 40 years of age had lower scores in domain V (p=0.044). People with HIV with a partner had lower scores in domains I (p=0.004) and V (p=0.013). Participants with less than eight years of schooling had lower scores in domain III (p=0.031). Those with children had lower scores in domains I (p=0.004), III (p=0.002), IV (p=0.002) and V (p<0.001) (Tables 2 and 3).

**Table 2:** Association of sociodemographic variables of people with HIV and the mean scores of domains I, II and III of the WHOQOL-HIV-Bref (N=190), Fortaleza, Ceará, Brazil, 2023

Variables	I. Physical	II. Psychological	III. Level of independence
Sex			
Female	80.24	80.05	80.60
Male	101.25	101.32	101.12
Mann-Whitney U test	2794.50	2784.50	2813.00
Significance	**0.018**	**0.017**	**0.021**
Age			
< 40 years	100.25	95.81	98.65
≥ 40 years	88.22	95.02	90.67
Mann-Whitney U test	3766.50	4276.50	3950.00
Significance	0.139	0.922	0.323
Marital status			
With partner	81.66	88.92	85.36
Without partner	104.34	94.86	99.50
Mann-Whitney U test	3054.50	3803.00	3436.50
Significance	**0.004**	0.449	0.070
Education			
> eight years	93.72	99.39	103.69
≤ eight years	97.43	91.27	86.59
Mann-Whitney U test	4328.50	4119.50	3693.50
Significance	0.641	0.307	**0.031**
Have children			
Yes	81.67	87.36	80.54
No	104.92	101.04	105.69
Mann-Whitney U test	3285.50	3724.00	3198.50
Significance	**0.004**	0.091	**0.002**
Number of people in the household			
> three people	84.59	75.92	78.86
≤ three people	99.40	102.49	101.44
Mann-Whitney U test	2954.50	2521.00	2668.00
Significance	0.101	**0.003**	**0.012**
Occupational status			
Employed	108.69^‡^	111.03^†^	112.27^†^
Retired	135.13^‡^	110.75^†.‡^	124.25^†.‡^
Leave/sick pay	72.35^†^	74.59^b^	72.09^‡^
Unemployed	77.54^†^	74.63^b^	72.41^‡^
Kruskal-Wallis test	18.114	20.552	25.728
Significance	**< 0.001**	**< 0.001**	**< 0.001**
Monthly income^*^			
≤ one minimum wage	79.64	83.89	79.85
> one minimum wage	104.34	101.97	104.22
Mann-Whitney U test	3069.50	3358.50	3084.00
Significance	**0.003**	**0.029**	**0.003**
Sexual orientation			
Heterosexual	84.20	89.94	78.83
Homosexual	102.91	96.44	108.96
Mann-Whitney U Test	3401.00	3963.50	2874.50
Significance	**0.017**	0.408	**< 0.001**

* Minimum wage - R$ 1,302.00, Brazil, 2023;

†,‡ Different symbols, different average positions (Dunn’s test).

**Table 3:** Association of sociodemographic variables of people with HIV and the mean scores of domains IV, V and VI of the WHOQOL-HIV-Bref (N=190), Fortaleza, Ceará, Brazil, 2023

Variables	IV. Social relationships	V. Environment	VI. Spirituality
Sex			
Female	82.02	78.38	80.13
Male	100.58	101.95	101.29
Mann-Whitney U test	2887.000	2698.000	2788.500
Significance	**0.036**	**0.008**	**0.017**
Age			
< 40 years	98.88	101.98	90.99
≥ 40 years	90.31	85.56	102.42
Mann-Whitney U test	3923.50	3567.00	3793.50
Significance	0.289	**0.044**	0.159
Marital status			
With partner	86.01	83.06	88.88
Without partner	98.65	102.50	94.91
Mann-Whitney U test	3503.50	3199.50	3799.00
Significance	0.105	**0.013**	0.441
Education			
> eight years	101.84	102.88	97.45
≤ eight years	88.60	87.47	93.38
Mann-Whitney U test	3877.00	3774.00	4311.50
Significance	0.094	0.053	0.608
Children			
Yes	80.92	76.27	88.26
No	105.43	108.61	100.43
Mann-Whitney U test	3228.00	2869.50	3793.00
Significance	**0.002**	**< 0.001**	0.132
Number of people in the household			
> three people	80.19	82.78	80.28
≤ three people	100.97	100.04	100.94
Mann-Whitney U test	2734.50	2864.00	2739.00
Significance	**0.021**	0.056	**0.022**
Occupational status			
Employed	112.59^†^	114.00^†^	103.91
Retired	138.25^†^	89.38^†.‡^	106.38
Leave/sick pay	54.76^‡^	53.85^‡^	97.47
Unemployed	75.62^‡^	76.48^‡^	80.50
Kruskal-Wallis test	30.817	29.590	7.485
Significance	**< 0.001**	**< 0.001**	0.058
Monthly income^*^			
≤ one minimum wage	73.01	68.39	90.87
>one minimum wage	108.04	110.61	98.08
Mann-Whitney U test	2618.50	2304.50	3833.00
Significance	**< 0.001**	**< 0.001**	0.383
Sexual orientation			
Heterosexual	85.54	82.61	91.46
Homosexual	101.41	104.71	94.73
Mann-Whitney U test	3531.50	3244.50	4112.50
Significance	**0.042**	**0.005**	0.677

^^*^^Minimum wage - R$ 1,302.00, Brazil, 2023;

†,‡ Different symbols, different average positions (Dunn’s test).

Those who lived with more than three people had lower scores in domains II (p=0.003), III (p=0.012), IV (p=0.021) and VI (p=0.022). People with HIV who were unemployed and on sick leave/benefit had lower scores than those who were employed and retired in domains I (p<0.001), II (p<0.001), III (p<0.001), IV (p<0.001) and V (p<0.001). Having an income lower than one minimum wage was associated with lower scores in domains I (p=0.003), II (p=0.029), III (p=0.003), IV (p<0.001) and V (p<0.001). Heterosexuals had lower scores than homosexuals in domains I (p=0.017), II (p<0.001), IV (p=0.042) and V (p=0.005) (Tables 2 and 3).

In the analysis of the mean scores of clinical and vulnerability variables of WHOQOL-HIV-Bref domains, people with HIV with a CD4+ T lymphocyte count lower than 350 cells/mm^3^ had a lower perceived QoL in domain I (p=0.014), and those with a detectable viral load had lower scores in domains I (p=0.016) and IV (p=0.016). Participants who did not accept positive HIV serology had lower scores in domains I (p<0.001), II (p=0.001), III (p<0.001), IV (p<0.001), V (p<0.003) and VI (p<0.032). People with HIV who suffered stigma/prejudice had a lower perceived QoL in domains I (p=0.005), II (p=0.010), III (p<0.020), IV (p<0.001) and V (p<0.001). Those who had been through the prison system had lower values in domains IV (p=0.007) and V (p=0.018). People with HIV who were dissatisfied with their healthcare monitoring had lower QoL scores in domains I (p=0.019), II (p=0.048) and V (p=0.005) (Tables 4 and 5).

**Table 4:** Association of clinical and vulnerability variables of people with HIV and mean scores of domains I, II and III of the WHOQOL-HIV-Bref (N=190), Fortaleza, Ceará, Brazil, 2023

Variables	I. Physical	II. Psychological	III. Level of independence
HIV+ diagnosis time			
< eight years	96.71	93.45	95.64
≥ eight years	94.21	97.68	95.35
Mann-Whitney U test	4389.00	4307.00	4494.50
Significance	0.752	0.594	0.971
Time on antiretroviral therapy			
< eight years	93.67	92.66	94.47
≥ eight years	97.91	99.24	96.85
Mann-Whitney U test	4230.00	4121.50	4317.00
Significance	0.596	0.412	0.765
CD4+ T lymphocyte count			
> 350 cells/mm^3^	99.66	94.70	97.52
≤ 350 cells/mm^3^	72.40	99.91	84.26
Mann-Whitney U test	1664.50	2206.50	2008.50
Significance	**0.014**	0.637	0.227
Viral load			
Detectable	69.61	81.00	77.41
Undetectable	99.07	97.50	97.99
Mann-Whitney U test	1325.00	1587.00	1504.50
Significance	**0.016**	0.175	0.890
Accepts HIV diagnosis			
Yes	101.15	99.71	100.95
No	44.66	57.58	46.42
Mann-Whitney U test	658.50	904.00	692.00
Significance	**< 0.001**	**0.001**	**< 0.001**
Experiences stigma/prejudice			
Yes	72.52	74.18	75.99
No	97.92	97.36	96.75
Mann-Whitney U test	2255.00	2331.50	2414.50
Significance	**0.005**	**0.010**	**0.020**
Sexual relations for money or drugs			
Yes	91.13	80.71	95.69
No	96.13	97.64	95.47
Mann-Whitney U test	1887.00	1637.00	1987.50
Significance	0.675	0.157	0.986
Time in the prison system			
Yes	101.10	51.30	93.70
No	95.35	96.69	95.55
Mann-Whitney U test	434.50	241.50	453.50
Significance	0.817	0.067	0.940
Satisfaction with healthcare			
Yes	98.03	97.63	96.90
No	61.08	66.50	80.58
Mann-Whitney U test	703.00	773.50	956.50
Significance	**0.019**	**0.048**	0.306

**Table 5:** Association of clinical and vulnerability variables of people with HIV and mean scores of domains IV, V and VI of the WHOQOL-HIV-Bref (N=190), Fortaleza, Ceará, Brazil, 2023

Variables	IV. Social relationships	V. Environment	VI. Spirituality
Time since diagnosis			
< eight years	102.43	100.24	92.74
≥ eight years	88.12	90.45	98.43
Mann-Whitney U test	3829.00	4043.00	4238.00
Significance	0.07	0.218	0.473
Time on antiretroviral therapy			
< eight years	98.90	100.50	93.22
≥ eight years	91.02	88.92	98.51
Mann-Whitney U test	4060.50	3888.50	4181.50
Significance	0.323	0.150	0.509
CD4+ T lymphocyte count			
> 350 cells/mm^3^	97.32	96.96	97.52
≤ 350 cells/mm^3^	85.38	87.38	84.26
Mann-Whitney U test	2041.00	2099.00	2008.50
Significance	0.227	0.386	0.229
Viral load			
Detectable	69.89	83.04	85.43
Undetectable	99.03	97.22	96.89
Mann-Whitney U test	1331.50	1634.00	1689.00
Significance	**0.016**	0.245	0.346
Accepts HIV diagnosis			
Yes	100.21	99.41	98.34
No	53.13	60.29	69.92
Mann-Whitney U test	819.50	955.50	1138.50
Significance	**< 0.001**	**0.003**	**0.032**
Experiences stigma/prejudice			
Yes	65.49	64.28	79.86
No	100.30	100.71	95.44
Mann-Whitney U test	1931.50	1876.00	2592.50
Significance	**< 0.001**	**< 0.001**	0.081
Sexual intercourse for money or drugs			
Yes	75.35	75.44	100.46
No	98.41	98.40	94.78
Mann-Whitney U test	1508.50	1510.50	1873.00
Significance	0.053	0.055	0.634
Time in the prison system			
Yes	30.30	38.00	80.40
No	97.26	97.05	95.91
Mann-Whitney U test	136.50	175.00	387.00
Significance	**0.007**	**0.018**	0.531
Satisfaction with follow-up in health			
Yes	96.79	98.53	94.99
No	77.92	54.19	102.46
Mann-Whitney U test	922.00	613.50	1060.00
Significance	0.228	**0.005**	0.634

## DISCUSSION

This study assessed QoL of people with HIV and seroconcordant and serodiscordant sexual partners, finding that the majority had a better perceived QoL, with no significant difference between groups. This finding may be due to ART, which enables viral suppression, reduces mortality and increases life expectancy^(3,4)^. It is important to note that immunological control is important for these people to have sexual relations, especially due to the risk of transmission of the virus^(14)^, in addition to stigma related to the infection, which can negatively affect QoL^(13)^.

The perspective that an undetectable viral load is the same as an untransmittable viral load indicates that a person with HIV with a suppressed viral load does not sexually transmit the virus, but there is little knowledge of the percentage of individuals at the population level who sustain long-term viral suppression^(7,8)^. Research showed that, of those suppressed, 54.3% maintained viral suppression and 33.6% had virological failure during the study, and among those with virological failure, 82.6% did so six or more months after consecutive suppression^(8)^. This supports the need for continuous viral load monitoring, encouragement of ART and guidance on combination prevention methods^(8,9)^.

Even without significant differences in QoL of people with HIV who are seroconcordant and serodiscordant, in the analysis of the total sample, some variables were associated in WHOQOL-HIV-Bref domains. Females had lower scores in all domains, indicating gender issues, in which women encounter barriers in various aspects of life, especially in socioeconomic terms^(27)^, with lower income and food insecurity, which negatively influence QoL^(28)^. Other variables associated with lower QoL scores, except for domain VI (spirituality), were unemployment and income below the minimum wage, in agreement with another study, which observed that unemployment interferes with QoL of people with HIV^(29)^. As for the exception of domain VI, it may be associated with the importance of religious support, since religiosity has a positive influence on QoL^(29,30)^.

In domain I (physical), lower scores were found in people with HIV who had a partner, children and heterosexuals. The literature shows that having a partner can be a source of social support, as married people tend to have better QoL scores^(16-21)^. Furthermore, domain I also encompasses satisfaction with sleep, which may be impaired in participants with children, with sleep disorders being recurrent in this population^(31)^. Regarding domain II (psychological), the variable living with more than three people was associated with lower QoL scores. This may be due to lower per capita income, as this domain addresses the perception of how much one enjoys life^(29)^.

In domain III (level of independence), having less than eight years of education, having children, living with more than three people and being heterosexual were related to lower QoL scores. Among the aspects addressed in this domain is the ability to work, which may be impaired by lower education, unemployment and difficulties in advancing in the job market, especially due to stigma due to HIV^(32,33)^. Despite the lower scores in domain III among those with children and who lived with more than three people in the household, another study found no difference in QoL of people with HIV with and without children as well as among those with a joint or nuclear family^(34)^.

In domain IV (social relationships), having children, living with three or more people in the household and being heterosexual were variables associated with lower QoL scores. This domain addresses satisfaction with personal relationships and sexual life. HIV infection has a negative impact on the lives of people and their families, so much so that research with HIV+ women observed family separation^(35)^. Concerning sexuality, despite the lower QoL scores among heterosexuals in this domain, same-sex relationships between people with HIV still face prejudice, due to stigma and intersections of gender, sexual orientation and culture^(36)^. Homophobia delays diagnosis and reduces testing in those with higher levels of homophobia and unknown HIV status, with testing being higher among those without prejudice in same-sex relationships^(37)^.

In domain V (environment), being over 40 years old, being heterosexual, having a partner and children were variables associated with lower QoL scores. Older people with HIV may have fewer opportunities to enter the job market, and younger people are more likely to be employed^(33)^. Lower scores among heterosexuals in domain V may be related to low availability of information and access to healthcare services, since as they do not belong to vulnerable populations, they are not the focus of HIV prevention actions^(38)^. As for having a partner and children, as domain V also addresses security in daily life, financial resources and leisure, socioeconomic issues stand out^(29)^, as financial support contributes to adherence to ART, healthy eating and access to healthcare^(39)^.

In domain VI (spirituality), those who lived with three or more people in the household had lower scores. Since this domain addresses fear of the future, these scores may be due to concerns about the family unit, as people with HIV who have children are afraid and stressed^(35)^. Not accepting diagnosis was related to lower QoL scores; in this regard, studies show that religious support contributes to coping with HIV infection^(40)^, positively influencing QoL^(30)^. Not accepting positive HIV serology was associated with lower scores in all domains of the WHOQOL-HIV-Bref. Acceptance of diagnosis is important for adherence to ART and is related to satisfactory QoL^(41)^, while denial may be accompanied by negative psychosocial reactions^(2)^. People with HIV who suffered stigma had lower scores in almost all QoL domains, except for VI (spirituality). Psychological events associated with HIV can occur due to stigma, which negatively influences QoL^(13,18)^, highlighting the importance of social support^(17)^.

In domain I, people with HIV with lower CD4+ T lymphocyte counts, detectable viral load and dissatisfied with health monitoring had lower scores, which shows the importance of immunological control with ART^(3,4)^. In domain II, lower satisfaction with health monitoring was related to lower scores. By addressing psychological aspects, such as personal satisfaction and frequency of negative feelings, there is occurrence of depressive events in people with HIV^(42)^, which can increase risk behaviors^(43)^. Regarding dissatisfaction with healthcare services, decentralizing care increases user satisfaction and improves adherence to ART^(44)^.

Domain III had lower scores for those who did not accept diagnosis and suffered stigma. This domain assesses the perceived need for treatment, showing that non-acceptance of the disease and delay in treatment should be addressed, since adherence to ART is affected by stigma^(39)^, and those who suffer stigma have more problems in the job market^(32,33)^. In domains IV and V, people with HIV with a detectable viral load and time in the prison system had lower QoL scores, inferring that stigma and prison history hinder access to healthcare^(45)^. There is an increased risk of HIV infection in people with a prison history, as access to ART and preventive measures is a challenge in these institutions^(46)^.

Finally, these data show the importance of comprehensive care for people with HIV. Monitoring laboratory tests and achieving viral load and CD4+ T lymphocyte count targets are essential activities performed by the health team. However, assessing people with HIV in a global manner can provide information that can guide health education practices for the well-being of these individuals and those with whom they interact.

### Study limitations

The COVID-19 pandemic was a limitation for carrying out the study, which interfered with data collection due to restrictions and changes in work dynamics in healthcare services.

### Contributions to nursing, health, or public policy

The research provided data to understand the different factors that influence QoL of people with HIV. Satisfaction with health monitoring stood out, which shows the importance of follow-up consultations by the multidisciplinary team, especially nurses, who play a key role in health education practices. Furthermore, the results of this study can help in the development of individual and collective interventions capable of improving QoL as well as guiding participants regarding self-care and combined HIV prevention strategies.

## CONCLUSIONS

Most people with HIV had a satisfactory QoL, with no difference between seroconcordant and serodiscordant individuals. There was a trend toward statistical significance in domain III (level of independence), in which those with a serodiscordant partner had a more intermediate perceived QoL compared to seroconcordant individuals. Being female and not accepting diagnosis were associated with a more unsatisfactory QoL. Those who were unemployed, on sick leave/benefit, with an income lower than one minimum wage and who suffered stigma had lower QoL scores in almost all domains, except spirituality/religion/beliefs. Heterosexuals, people over 40 years old, who lived with a partner, with less than eight years of education, who had children and more than three people in the household had lower scores in several domains. People with HIV with CD4+ T lymphocyte counts below 350 cells/mm^3^, detectable viral load, dissatisfied with healthcare and time in the prison system had a lower perceived QoL. These data show that although most participants had a higher perceived QoL, there are still aspects that can be assessed and addressed by nursing and other members of the multidisciplinary team, aiming at the well-being of people with HIV and care to avoid transmitting the virus.

## Data Availability

The research data are available within the article.
