# Supplementary material for: Quality of life of people with human immunodeficiency virus and seroconcordant and serodiscordant partnerships
Source: Rev Bras Enferm. 2025 Oct 3;78(Suppl 2):e20240569. doi: 10.1590/0034-7167-2024-0569 (PMC12500076; doi:10.1590/0034-7167-2024-0569)
Supplement: Translation

# Translation: Qualidade de vida das pessoas com vírus da imunodeficiência humana e parcerias soroconcordantes e sorodiscordantes

**DOI:** 10.1590/0034-7167-2024-0569pt

**Authors:** Larissa Rodrigues Siqueira, Gilmara Holanda da Cunha, Marcos Venícios de Oliveira Lopes, Maria Elisa Curado Gomes, Ane Kelly Lima Ramalho, Maiara Bezerra Dantas, Vanessa Sousa dos Santos, Giovanna Soares Lins

**Affiliations:** I Universidade Federal do Ceará. Fortaleza, Ceará, Brasil

**Keywords:** HIV, Qualidade de Vida, Parceiros Sexuais, Enfermagem, Promoção da Saúde.

## Abstract

**Objetivos:**

avaliar a qualidade de vida das pessoas com HIV com parcerias soroconcordantes e sorodiscordantes.

**Métodos:**

estudo transversal, realizado em três ambulatórios com 190 pessoas com HIV (95 com parcerias soroconcordantes e 95 sorodiscordantes), utilizando-se formulário de caracterização e instrumento de avaliação da qualidade de vida. Realizaram-se estatística descritiva e associações entre variáveis, considerando-se estatisticamente significante valor de p<0,05.

**Resultados:**

a maioria teve percepção superior da qualidade de vida, sem diferença entre grupos. O sexo feminino teve escores inferiores em todos os domínios. Variáveis associadas à qualidade de vida insatisfatória foram ter filhos, morar com mais de três pessoas, desemprego, renda menor que um salário mínimo, linfócitos TCD4+ menores que 350 células/mm^3^, carga viral detectável, não aceitar diagnóstico, estigma, passagem pelo sistema prisional e insatisfação com o acompanhamento em saúde.

**Conclusões:**

a percepção da qualidade de vida foi superior para a maioria dos soroconcordantes e sorodiscordantes, sem diferença entre grupos.

## INTRODUÇÃO

O diagnóstico da infecção pelo vírus da imunodeficiência humana (HIV) envolve inicialmente a aceitação da sorologia, que pode associar-se à culpa, medo e decepção^(1,2)^. O diagnóstico precoce possibilita o início da terapia antirretroviral (TARV), que proporciona a supressão viral, redução da mortalidade e aumento da sobrevida^(3-5)^. A carga viral indetectável é intransmissível^(6)^, mas podem ocorrer casos de falha da supressão sustentada e a carga viral tornar-se detectável novamente^(7)^, o que mostra a importância da sua monitorização^(8)^ e do uso dos métodos da prevenção combinada para reduzir a incidência do HIV^(9)^.

A adesão adequada à TARV melhora a qualidade de vida (QV) das pessoas com HIV^(3,4)^. A QV é a percepção do indivíduo sobre sua posição na vida, contexto de cultura e sistema de valores em relação aos seus objetivos, expectativas, padrões e preocupações^(10)^. Pode ser mensurada por meio de instrumentos, mas existe um específico para pessoas com HIV, que avalia a QV de forma multidimensional, por meio dos domínios físico, psicológico, nível de independência, relações sociais, meio ambiente, espiritualidade/religião/crenças pessoais^(10)^. A QV das pessoas com HIV pode ser prejudicada pelo estigma nas relações afetivas e sexuais, medo do abandono e rejeição por revelar o diagnóstico^(11-13)^. A sexualidade influencia a QV, pois a sorologia anti-HIV positiva pode diminuir o envolvimento afetivo, pelo risco de transmissão às parcerias^(14)^. Assim, as pessoas com HIV precisam de orientações sobre suas parcerias sexuais e a importância da carga viral indetectável, a fim de melhorar os aspectos da saúde sexual e a capacidade de revelar o diagnóstico^(15)^.

Ressalta-se a influência dos relacionamentos para a QV dessa população, pois ter um(a) companheiro(a) pode significar maior suporte social e melhor QV^(16)^. Também é importante avaliar a QV das pessoas com HIV com diferentes tipos de parcerias sexuais, sejam elas soroconcordantes ou sorodiscordantes, pois estudo identificou que a QV foi melhor no grupo sem o vírus, o que denota que as pessoas com HIV podem ter uma QV insatisfatória, quando comparadas à população geral^(12)^.

Considerando-se os avanços alcançados com a TARV^(3,4)^ e seus efeitos na QV^(14,16)^, torna-se relevante avaliar a QV das pessoas com HIV com parcerias soroconcordantes e sorodiscordantes. Apesar de haver estudos sobre a temática^(16-18)^ e que comparam sorodiscordantes e soroconcordantes^(12,19)^, pesquisas atualizadas sobre a QV em diferentes regiões geográficas são importantes, especialmente após a pandemia de coronavírus-19 (COVID-19), que reestruturou alguns serviços de saúde^(20)^. Essas análises podem detectar os aspectos da vida mais afetados pela doença e ajudar os profissionais de saúde e formuladores de políticas públicas a direcionarem melhor suas ações^(21)^. Diante do exposto, a hipótese deste estudo é que as pessoas com HIV com parceria sorodiscordante tenham uma QV mais insatisfatória que as soroconcordantes.

## OBJETIVOS

Avaliar a QV de pessoas com HIV com parcerias sexuais soroconcordantes e sorodiscordantes.

## MÉTODOS

### Aspectos éticos

O projeto foi aprovado pelo Comitê de Ética em Pesquisa da Universidade Federal do Ceará e instituições coparticipantes. Seguiu-se a Resolução n° 466/2012 do Conselho Nacional de Saúde. Todos os participantes assinaram o Termo de Consentimento Livre e Esclarecido (TCLE).

### Desenho, período, local do estudo, população e amostra

Trata-se de estudo transversal, segundo as recomendações do *STrengthening the Reporting of OBservational studies in Epidemiology* ^(22)^. A coleta de dados ocorreu de janeiro de 2022 a dezembro de 2023. O estudo foi realizado em três ambulatórios públicos de infectologia em Fortaleza, Ceará, Brasil, os quais são referência no atendimento às pessoas com HIV na região Nordeste. A população foi composta por pessoas com HIV que tivessem parceria fixa ou eventual, soroconcordante ou sorodiscordante. Para cálculo amostral, considerou-se a variação na pontuação do instrumento de QV^(10)^ entre pessoas com HIV com parceria soroconcordante e sorodiscordante, nível de confiança de 95%, poder de 80% e diferença de 7% nas pontuações entre os dois grupos, utilizando-se a fórmula abaixo para comparação de médias:


$$\begin{array}{c} N=\left(\sigma_{1}^{2}+\sigma_{2}^{2}\right)\cdot {\left(Z_{a/2}+Z_{1-\beta }\right)}^{2}\\ {\left(\mu_{2}-\mu_{1}\right)}^{2} \end{array}$$

A amostra foi de 90 participantes por grupo. Devido à assimetria de valores obtidos em escalas, foi utilizada a correção de Pitman, calculando-se 95 participantes por grupo, com o total de 190 pessoas com HIV. A amostragem foi não probabilística por conveniência. Critérios de inclusão abrangeram pessoas com HIV de ambos os sexos, maiores de 18 anos, sexualmente ativas, com parceria sexual fixa ou eventual, soroconcordante ou sorodiscordante. Critérios de exclusão abrangeram gestantes.

### Protocolo do estudo

As pessoas com HIV foram convidadas a participar da pesquisa enquanto aguardavam a consulta médica que ocorre de forma semestral. O estudo foi explanado, e aqueles que concordaram em participar assinaram o TCLE. A entrevista foi realizada em consultório privativo, com duração média de 30 minutos, utilizando-se dois instrumentos. O Formulário de Caracterização Sociodemográfica, Clínica, Epidemiológica e de Vulnerabilidade para Pessoas com HIV^(23)^ incluiu as seguintes variáveis: idade; sexo; escolaridade; estado civil; situação ocupacional; número de pessoas no domicílio; renda mensal familiar; tempo de diagnóstico e de TARV; orientação sexual; reside com parceria; sorologia da parceria; número de filhos; contagem de linfócitos T CD4+; carga viral; aceitação do diagnóstico; vivência de estigma; passagem pelo sistema prisional; e satisfação com o serviço de saúde.

O instrumento de avaliação da QV, o *World Health Organization Quality of Life Instrument* - HIV/aids module (WHOQOL-HIV-Bref), foi desenvolvido por pesquisadores vinculados à Organização Mundial da Saúde na língua portuguesa validada no Brasil^(10)^. O questionário original tem 120 perguntas, 29 facetas e uma faceta sobre QV geral e percepção geral de saúde, e seis domínios: I. Físico (dor, desconforto, energia, fadiga, sono, descanso); II. Psicológico (sentimentos positivos e negativos, cognição, autoestima, imagem corporal, aparência); III. Nível de independência (mobilidade, atividades de vida diária, dependência de medicação e tratamentos, aptidão ao trabalho); IV. Relações sociais (relacionamentos pessoais, apoio social, atividade sexual); V. Meio ambiente (segurança física, moradia, finanças, acesso à saúde e assistência social, capacidade de adquirir informações e aprender novas habilidades, lazer, ambiente físico, transporte); VI. Espiritualidade/religião/crenças (perdão, culpa, preocupações sobre o futuro, morte e morrer)^(10)^.

Neste estudo, utilizou-se a versão abreviada do instrumento, o WHOQOL-HIV-Bref, com 31 perguntas e seis domínios, e as facetas sobre QV geral e percepção geral de saúde^(10)^. As questões são pontuadas em escala tipo Likert de 5 pontos, em que 1 indica percepção baixa e negativa, e 5, a percepção alta e positiva acerca da QV. Os escores variam de 4 a 20 pontos, refletindo a pior e a melhor percepção da QV, respectivamente. A QV das pessoas com HIV foi estratificada em insatisfatória e satisfatória, conforme a pergunta 1 do WHOQOL-HIV-Bref^(10)^. A caracterização da percepção da QV por escores dos domínios ocorreu da seguinte forma: 4 - 9,9 (inferior); 10 - 14,9 (intermediária); e 15 - 20 (superior)^(24,25)^. Escores mais baixos indicam uma percepção mais inferior da QV^(26)^.

### Análise dos dados e estatística

A análise exploratória constou de testes estatísticos descritivos, frequências absolutas e relativas, médias, medianas, e desvios padrão. O teste de normalidade de Shapiro-Wilk foi utilizado para verificar a distribuição de escores. Dependendo dos resultados de homogeneidade de variância e aderência à distribuição normal, aplicaram-se, para casos de não normalidade, o teste de Mann-Whitney, para comparar postos médios entre variáveis constituídas de dois grupos, o teste de Kruskal-Wallis, para aquelas com mais de dois grupos, e o teste de Dunn, para comparações *post-hoc*. Para associar as variáveis e os escores dos domínios do WHOQOL-HIV-Bref, aplicaram-se o teste qui-quadrado, teste exato Fisher ou teste de Fisher-Freeman-Halton, e o teste z, para comparações *post-hoc*. O nível de significância foi estabelecido em 5%, considerando-se estatisticamente significante o valor de p<0,05. O *software Statistical Package for the Social Sciences,* versão 23.0, foi utilizado para análises.

## RESULTADOS

Da amostra total de 190 pessoas com HIV, a maioria era do sexo masculino (138; 72,7%), com cor da pele autorreferida parda (67,3%), casada/união estável (54,2%), sem filhos (59,4%), com emprego (55,2%), renda mensal maior que um salário mínimo (64,2%), heterossexual (51,6%), da categoria de exposição sexual (93,1%) e morava com parceria (54,8%). A maioria tinha contagem de linfócitos T CD4+ maior que 350 células/mm^3^ (84,8%), carga viral indetectável (87,9%), aceitava o diagnóstico (90%) e estava satisfeita com o acompanhamento em saúde (93,1%). Alguns tinham tempo de diagnóstico (51,6%) e de TARV (56,9%) menor que oito anos, sofriam estigma/preconceito (24,2%), mantinham relação sexual por dinheiro/drogas (12,7%) e tinham passagem pelo sistema prisional (2,6%).

A idade variou de 21 a 65 anos, com médias e desvios padrão para soroconcordantes de 39,95 ± 10,61, e sorodiscordantes, de 35,82 ± 10,22. O número de anos de estudo variou de zero a 20, com medianas e intervalos interquartis para de 10 e 6, para soconcordantes, e 8 e 5, para sorodiscordantes. A renda mensal variou de R$150,00 a R$20.000,00, com medianas e intervalos interquartis para soconcordantes de 1.818,00 e 1.800,00, e para sorodiscordantes, de 2.000,00 e 2.400,00.

Os grupos de pessoas com HIV com parceria soroconcordante e sorodiscordante foram semelhantes em todos os domínios do WHOQOL-HIV-Bref, com dados não aderentes à distribuição normal (p<0,05). As medianas nos domínios para soroconcordantes foram 15,00 (I), 16,00 (II), 15,00 (III), 16,00 (IV), 14,50 (V) e 17,00 (VI). Para sorodiscordantes, esses valores foram 15,00 (I), 16,00 (II), 14,00 (III), 16,00 (IV), 15,00 (V) e 17,00 (VI). A maioria das pessoas com HIV teve uma percepção superior da QV, com análise similar entre aqueles com parceria sexual soroconcordante e sorodiscordante, não sendo verificada diferença estatisticamente significante. Na classificação da QV por domínio, não houve diferença entre pessoas com HIV soroconcordantes e sorodiscordantes, apenas uma tendência de significância estatística no domínio III (nível de independência), no qual os sorodiscordantes tiveram uma percepção mais intermediária da QV, comparados aos soroconcordantes (Tabela 1).

**Tabela 1:** Qualidade de vida das pessoas com HIV com parceria soroconcordante e sorodiscordante, valores dos postos médios por domínio (N=190), Fortaleza, Ceará, Brasil, 2023

Domínios do WHOQOL-HIV-Bref	Soroconcordante	Sorodiscordante	X^2^	gl	U	Valor de *p*
	n (%)	n (%)				
Postos médios						
Domínio I	89,57	101,43			3949,50	0,136
Domínio II	88,93	102,07			3888,00	0,098
Domínio III	94,42	96,58			4410,00	0,785
Domínio IV	88,18	102,82			3817,00	0,064
Domínio V	88,07	102,93			3806,50	0,062
Domínio VI	97,66	93,34			4307,50	0,586
Classificação da qualidade de vida						
I - Físico						
Inferior	9 (9,5)	6 (6,3)	1,0	2		0,587
Intermediária	34 (35,8)	31 (32,6)				
Superior	52 (54,7)	58 (61,1)				
II - Psicológico						0,506^§^
Inferior	5 (5,3)	3 (3,2)	-	-		
Intermediária	33 (34,7)	27 (28,4)				
Superior	57 (60,0)	65 (68,4)				
III - Nível de independência						0,058^§^
Inferior	7 (7,4)	2 (2,1)	-	-		
Intermediária	34 (35,8)	48 (50,5)				
Superior	54 (56,8)	45 (47,4)				
IV - Relações sociais						0,455^§^
Inferior	2 (2,1)	1 (1,1)	-	-		
Intermediária	34 (35,8)	27 (28,4)				
Superior	59 (62,1)	67 (70,5)				
V - Meio ambiente						0,271^§^
Inferior	1 (1,1)	2 (2,1)	-	-		
Intermediária	49 (51,5)	38 (40,0)				
Superior	45 (47,4)	55 (57,9)				
VI - Espiritualidade/religião/crenças						0,145^§^
Inferior	5 (5,3)	1 (1,1)	-	-		
Intermediária	26 (27,3)	34 (35,7)				
Superior	64 (67,4)	60 (63,2)				

X^2^ - teste qui-quadrado; gl - graus de liberdade; U - teste U de Mann-Whitney;

§ teste exato de Fisher.

Como não houve diferença entre os grupos de soroconcordantes e sorodiscordantes, realizou-se uma análise com a amostra total por domínio do WHOQOL-HIV-Bref. Segundo os escores, considerando-se as 190 pessoas com HIV, teve-se, respectivamente, a mediana, intervalo interquartil e valor de p do: domínio I (15,00; 5,00; p<0,001); domínio II (16,00; 4,00; p<0,001); domínio III (15,00; 3,00; p<0,001); domínio IV (16,00; 3,00; p<0,001); domínio V (15,00; 2,60; p<0,002); domínio VI (17,00; 5,00; p<0,001). Os escores por domínio indicaram que a maioria tinha percepção superior da QV. Na análise da QV geral, pela primeira pergunta do instrumento, somente 21,6% da amostra teve QV insatisfatória (mediana: 4,00).

Na análise dos postos médios das variáveis sociodemográficas associadas aos domínios do WHOQOL-HIV-Bref, o sexo feminino teve escores inferiores nos domínios I (físico) (p=0,018), II (psicológico) (p=0,017), III (nível de independência) (p=0,021), IV (relações sociais) (p=0,036), V (meio ambiente) (p=0,008) e VI (espiritualidade/religião/crenças) (p=0,017). Aqueles com mais de 40 anos tiveram menores escores no domínio V (p=0,044). As pessoas com HIV com companheiro(a) tiveram escores inferiores nos domínios I (p=0,004) e V (p=0,013). Participantes com menos de oito anos de estudo tiveram escores inferiores no domínio III (p=0,031). Aqueles com filhos tiveram escores mais baixos nos domínios I (p=0,004), III (p=0,002), IV (p=0,002) e V (p<0,001) (Tabelas 2 e 3).

**Tabela 2:** Associação das variáveis sociodemográficas das pessoas com HIV e os postos médios dos domínios I, II e III do WHOQOL-HIV-Bref (N=190), Fortaleza, Ceará, Brasil, 2023

Variáveis	I. Físico	II. Psicológico	III. Nível de independência
Sexo			
Feminino	80,24	80,05	80,60
Masculino	101,25	101,32	101,12
Teste U de Mann-Whitney	2794,50	2784,50	2813,00
Significância	**0,018**	**0,017**	**0,021**
Idade			
< 40 anos	100,25	95,81	98,65
≥ 40 anos	88,22	95,02	90,67
Teste U de Mann-Whitney	3766,50	4276,50	3950,00
Significância	0,139	0,922	0,323
Estado civil			
Com companheiro(a)	81,66	88,92	85,36
Sem companheiro(a)	104,34	94,86	99,50
Teste U de Mann-Whitney	3054,50	3803,00	3436,50
Significância	**0,004**	0,449	0,070
Escolaridade			
> oito anos	93,72	99,39	103,69
≤ oito anos	97,43	91,27	86,59
Teste U de Mann-Whitney	4328,50	4119,50	3693,50
Significância	0,641	0,307	**0,031**
Tem filhos			
Sim	81,67	87,36	80,54
Não	104,92	101,04	105,69
Teste U de Mann-Whitney	3285,50	3724,00	3198,50
Significância	**0,004**	0,091	**0,002**
Número de pessoas no domicílio			
> três pessoas	84,59	75,92	78,86
≤ três pessoas	99,40	102,49	101,44
Teste U de Mann-Whitney	2954,50	2521,00	2668,00
Significância	0,101	**0,003**	**0,012**
Situação ocupacional			
Empregado	108,69^‡^	111,03^†^	112,27^†^
Aposentado	135,13^‡^	110,75^†,‡^	124,25^†,‡^
Licença/auxílio-doença	72,35^†^	74,59^b^	72,09^‡^
Desempregado	77,54^†^	74,63^b^	72,41^‡^
Teste de Kruskal-Wallis	18,114	20,552	25,728
Significância	**< 0,001**	**< 0,001**	**< 0,001**
Renda mensal^^*^^			
≤ um salário mínimo	79,64	83,89	79,85
> um salário mínimo	104,34	101,97	104,22
Teste U de Mann-Whitney	3069,50	3358,50	3084,00
Significância	**0,003**	**0,029**	**0,003**
Orientação sexual			
Heterossexual	84,20	89,94	78,83
Homossexual	102,91	96,44	108,96
Teste U de Mann-Whitney	3401,00	3963,50	2874,50
Significância	**0,017**	0,408	**< 0,001**

^^*^^Salário mínimo - R$ 1.302,00, Brasil, 2023;

†,‡ Símbolos diferentes, postos médios diferentes (teste de Dunn).

**Tabela 3:** Associação das variáveis sociodemográficas das pessoas com HIV e os postos médios dos domínios IV, V e VI do WHOQOL-HIV-Bref (N=190), Fortaleza, Ceará, Brasil, 2023

Variáveis	IV. Relações sociais	V. Meio ambiente	VI. Espiritualidade
Sexo			
Feminino	82,02	78,38	80,13
Masculino	100,58	101,95	101,29
Teste U de Mann-Whitney	2887,000	2698,000	2788,500
Significância	**0,036**	**0,008**	**0,017**
Idade			
< 40 anos	98,88	101,98	90,99
≥ 40 anos	90,31	85,56	102,42
Teste U de Mann-Whitney	3923,50	3567,00	3793,50
Significância	0,289	**0,044**	0,159
Estado civil			
Com companheiro(a)	86,01	83,06	88,88
Sem companheiro(a)	98,65	102,50	94,91
Teste U de Mann-Whitney	3503,50	3199,50	3799,00
Significância	0,105	**0,013**	0,441
Escolaridade			
> oito anos	101,84	102,88	97,45
≤ oito anos	88,60	87,47	93,38
Teste U de Mann-Whitney	3877,00	3774,00	4311,50
Significância	0,094	0,053	0,608
Filhos			
Sim	80,92	76,27	88,26
Não	105,43	108,61	100,43
Teste U de Mann-Whitney	3228,00	2869,50	3793,00
Significância	**0,002**	**< 0,001**	0,132
Pessoas no domicílio			
> três pessoas	80,19	82,78	80,28
≤ três pessoas	100,97	100,04	100,94
Teste U de Mann-Whitney	2734,50	2864,00	2739,00
Significância	**0,021**	0,056	**0,022**
Situação ocupacional			
Empregado	112,59^†^	114,00^†^	103,91
Aposentado	138,25^†^	89,38^†,‡^	106,38
Licença/auxílio-doença	54,76^‡^	53,85^‡^	97,47
Desempregado	75,62^‡^	76,48^‡^	80,50
Teste de Kruskal-Wallis	30,817	29,590	7,485
Significância	**< 0,001**	**< 0,001**	0,058
Renda mensal^^*^^			
≤ um salário mínimo	73,01	68,39	90,87
>um salário mínimo	108,04	110,61	98,08
Teste U de Mann-Whitney	2618,50	2304,50	3833,00
Significância	**< 0,001**	**< 0,001**	0,383
Orientação sexual			
Heterossexual	85,54	82,61	91,46
Homossexual	101,41	104,71	94,73
Teste U de Mann-Whitney	3531,50	3244,50	4112,50
Significância	**0,042**	**0,005**	0,677

^^*^^Salário mínimo - R$ 1.302,00, Brasil, 2023;

†,‡ Símbolos diferentes, postos médios diferentes (teste de Dunn).

Os que moravam com mais de três pessoas tiveram escores inferiores nos domínios II (p=0,003), III (p=0,012), IV (p=0,021) e VI (p=0,022). As pessoas com HIV desempregadas e de licença/auxílio-doença tiveram escores inferiores aos empregados e aposentados nos domínios I (p<0,001), II (p<0,001), III (p<0,001), IV (p<0,001) e V (p<0,001). Ter renda menor que um salário mínimo foi associado a menores escores nos domínios I (p=0,003), II (p=0,029), III (p=0,003), IV (p<0,001) e V (p<0,001). Heterossexuais tiveram menores escores que os homossexuais nos domínios I (p=0,017), II (p<0,001), IV (p=0,042) e V (p=0,005) (Tabelas 2 e 3).

Na análise dos postos médios das variáveis clínicas e de vulnerabilidade dos domínios do WHOQOL-HIV-Bref, as pessoas com HIV com contagem de linfócitos T CD4+ menor que 350 células/mm^3^ tiveram percepção mais inferior da QV no domínio I (p=0,014), e aquelas com carga viral detectável tiveram escores mais baixos nos domínios I (p=0,016) e IV (p=0,016). Participantes que não aceitavam a sorologia anti-HIV positiva tiveram escores inferiores nos domínios I (p<0,001), II (p=0,001), III (p<0,001), IV (p<0,001), V (p<0,003) e VI (p<0,032). As pessoas com HIV que sofriam estigma/preconceito tiveram percepção mais inferior da QV nos domínios I (p=0,005), II (p=0,010), III (p<0,020), IV (p<0,001) e V (p<0,001). Quem passou pelo sistema prisional teve menores valores nos domínios IV (p=0,007) e V (p=0,018). Pessoas com HIV insatisfeitas com o acompanhamento em saúde tiveram escores inferiores de QV nos domínios I (p=0,019), II (p=0,048) e V (p=0,005) (Tabelas 4 e 5).

**Tabela 4:** Associação das variáveis clínicas e de vulnerabilidade das pessoas com HIV e postos médios dos domínios I, II e III do WHOQOL-HIV-Bref (N=190), Fortaleza, Ceará, Brasil, 2023

Variáveis	I. Físico	II. Psicológico	III. Nível de independência
Tempo de diagnóstico HIV+			
< oito anos	96,71	93,45	95,64
≥ oito anos	94,21	97,68	95,35
Teste U de Mann-Whitney	4389,00	4307,00	4494,50
Significância	0,752	0,594	0,971
Tempo de terapia antirretroviral			
< oito anos	93,67	92,66	94,47
≥ oito anos	97,91	99,24	96,85
Teste U de Mann-Whitney	4230,00	4121,50	4317,00
Significância	0,596	0,412	0,765
Contagem de linfócitos T CD4+			
> 350 células/mm^3^	99,66	94,70	97,52
≤ 350 células/mm^3^	72,40	99,91	84,26
Teste U de Mann-Whitney	1664,50	2206,50	2008,50
Significância	**0,014**	0,637	0,227
Carga viral			
Detectável	69,61	81,00	77,41
Indetectável	99,07	97,50	97,99
Teste U de Mann-Whitney	1325,00	1587,00	1504,50
Significância	**0,016**	0,175	0,890
Aceita o diagnóstico de HIV			
Sim	101,15	99,71	100,95
Não	44,66	57,58	46,42
Teste U de Mann-Whitney	658,50	904,00	692,00
Significância	**< 0,001**	**0,001**	**< 0,001**
Sofre estigma/preconceito			
Sim	72,52	74,18	75,99
Não	97,92	97,36	96,75
Teste U de Mann-Whitney	2255,00	2331,50	2414,50
Significância	**0,005**	**0,010**	**0,020**
Relação sexual por dinheiro ou drogas			
Sim	91,13	80,71	95,69
Não	96,13	97,64	95,47
Teste U de Mann-Whitney	1887,00	1637,00	1987,50
Significância	0,675	0,157	0,986
Passagem pelo sistema prisional			
Sim	101,10	51,30	93,70
Não	95,35	96,69	95,55
Teste U de Mann-Whitney	434,50	241,50	453,50
Significância	0,817	0,067	0,940
Satisfação com acompanhamento em saúde			
Sim	98,03	97,63	96,90
Não	61,08	66,50	80,58
Teste U de Mann-Whitney	703,00	773,50	956,50
Significância	**0,019**	**0,048**	0,306

**Tabela 5:** Associação das variáveis clínicas e de vulnerabilidade das pessoas com HIV e postos médios dos domínios IV, V e VI do WHOQOL-HIV-Bref (N=190), Fortaleza, Ceará, Brasil, 2023

Variáveis	IV. Relações sociais	V. Meio ambiente	VI. Espiritualidade
Tempo de diagnóstico			
< oito anos	102,43	100,24	92,74
≥ oito anos	88,12	90,45	98,43
Teste U de Mann-Whitney	3829,00	4043,00	4238,00
Significância	0,07	0,218	0,473
Tempo de terapia antirretroviral			
< oito anos	98,90	100,50	93,22
≥ oito anos	91,02	88,92	98,51
Teste U de Mann-Whitney	4060,50	3888,50	4181,50
Significância	0,323	0,150	0,509
Contagem de linfócitos T CD4^+^			
> 350 células/mm^3^	97,32	96,96	97,52
≤ 350 células/mm^3^	85,38	87,38	84,26
Teste U de Mann-Whitney	2041,00	2099,00	2008,50
Significância	0,227	0,386	0,229
Carga viral			
Detectável	69,89	83,04	85,43
Indetectável	99,03	97,22	96,89
Teste U de Mann-Whitney	1331,50	1634,00	1689,00
Significância	**0,016**	0,245	0,346
Aceita o diagnóstico de HIV			
Sim	100,21	99,41	98,34
Não	53,13	60,29	69,92
Teste U de Mann-Whitney	819,50	955,50	1138,50
Significância	**< 0,001**	**0,003**	**0,032**
Sofre estigma/preconceito			
Sim	65,49	64,28	79,86
Não	100,30	100,71	95,44
Teste U de Mann-Whitney	1931,50	1876,00	2592,50
Significância	**< 0,001**	**< 0,001**	0,081
Relação sexual por dinheiro ou drogas			
Sim	75,35	75,44	100,46
Não	98,41	98,40	94,78
Teste U de Mann-Whitney	1508,50	1510,50	1873,00
Significância	0,053	0,055	0,634
Passagem pelo sistema prisional			
Sim	30,30	38,00	80,40
Não	97,26	97,05	95,91
Teste U de Mann-Whitney	136,50	175,00	387,00
Significância	**0,007**	**0,018**	0,531
Satisfação com acompanhamento em saúde			
Sim	96,79	98,53	94,99
Não	77,92	54,19	102,46
Teste U de Mann-Whitney	922,00	613,50	1060,00
Significância	0,228	**0,005**	0,634

## DISCUSSÃO

Este estudo avaliou a QV das pessoas com HIV e parcerias sexuais soroconcordantes e sorodiscordantes, constatando-se que a maioria tinha percepção superior da QV, sem diferença significante entre os grupos. Esse achado pode decorrer da TARV, que possibilita a supressão viral, reduz a mortalidade e aumenta a expectativa de vida^(3,4)^. Ressalta-se que o controle imunológico é importante para que essas pessoas se relacionem sexualmente, sobretudo pelo risco de transmissão do vírus^(14)^, além do estigma relacionado à infecção, que pode afetar negativamente a QV^(13)^.

A perspectiva de que a carga viral indetectável seja igual à intransmissível indica que uma pessoa com HIV com carga viral suprimida não transmite sexualmente o vírus, porém há pouco conhecimento do percentual de indivíduos em nível populacional que sustentam a supressão viral a longo prazo^(7,8)^. Pesquisa mostrou que, dos suprimidos, 54,3% mantinham a supressão viral e 33,6% tiveram falência virológica durante o estudo, e entre aqueles com falência virológica, 82,6% o fizeram seis ou mais meses após supressão consecutiva^(8)^. Isso apoia a necessidade de monitorização contínua da carga viral, incentivo à TARV e orientação dos métodos de prevenção combinada^(8,9)^.

Mesmo sem diferença significante na QV das pessoas com HIV soroconcordantes e sorodiscordantes, na análise da amostra total, algumas variáveis tiveram associação nos domínios do WHOQOL-HIV-Bref. O sexo feminino teve escores mais baixos em todos os domínios, apontando questões de gênero, em que as mulheres encontram barreiras em diversos aspectos da vida, sobretudo no socioeconômico^(27)^, com menor renda e insegurança alimentar, que influenciam negativamente a QV^(28)^. Outras variáveis associadas aos escores inferiores da QV, exceto pelo domínio VI (espiritualidade), foram o desemprego e renda menor que um salário mínimo, concordando com outro estudo, o qual observou que o desemprego interfere na QV das pessoas com HIV^(29)^. Quanto à exceção do domínio VI, pode estar associado à importância do amparo religioso, visto que a religiosidade tem influência positiva na QV^(29,30)^.

No domínio I (físico), ocorreram escores inferiores nas pessoas com HIV com companheiro(a), filhos e heterossexuais. A literatura mostra que ter um(a) companheiro(a) pode ser um suporte social, pois os casados tendem a apresentar melhores escores de QV^(16-21)^. Ademais, o domínio I também engloba a satisfação com o sono, o qual pode estar prejudicado nos participantes com filhos, sendo os distúrbios do sono recorrentes nesse público^(31)^. Quanto ao domínio II (psicológico), a variável morar com mais de três pessoas teve associação com escores inferiores de QV. Isso pode ocorrer pela menor renda *per capita*, pois esse domínio aborda a percepção do quanto se aproveita a vida^(29)^.

No domínio III (nível de independência), ter menos de oito anos de estudo, ter filhos, morar com mais de três pessoas e ser heterossexual teve relação com escores inferiores de QV. Entre os aspectos abordados nesse domínio, tem-se a capacidade de trabalhar, que pode estar prejudicada pela menor escolaridade, desemprego e dificuldades de ascensão no mercado de trabalho, sobretudo pelo estigma devido ao HIV^(32,33)^. Apesar dos menores escores no domínio III entre aqueles com filhos e que residiam com mais de três pessoas no domicílio, outro estudo não verificou diferença na QV das pessoas com HIV com e sem filhos, assim como entre aquelas com família conjunta ou nuclear^(34)^.

No domínio IV (relações sociais), ter filhos, viver com três ou mais pessoas no domicílio e ser heterossexual foram variáveis associadas a escores inferiores da QV. Esse domínio aborda a satisfação com as relações pessoais e vida sexual. A infecção pelo HIV tem impacto negativo na vida das pessoas e suas famílias, de forma que pesquisa com mulheres HIV+ observou a separação familiar^(35)^. Quanto à sexualidade, apesar dos escores inferiores da QV entre heterossexuais nesse domínio, as relações entre pessoas com HIV do mesmo sexo ainda enfrentam preconceitos, devido ao estigma e interseções de gênero, orientação sexual e cultura^(36)^. A homofobia retarda o diagnóstico e reduz a testagem naqueles com maior nível de homofobia e sorologia desconhecida para o HIV, sendo a testagem maior entre aqueles sem preconceito nas relações entre pessoas do mesmo sexo^(37)^.

No domínio V (meio ambiente), ter mais de 40 anos, ser heterossexual, ter companheiro(a) e filhos foram variáveis associadas a escores inferiores de QV. As pessoas com HIV de mais idade podem ter menos oportunidades de inserção no mercado de trabalho, e os mais jovens têm maior probabilidade para vínculo empregatício^(33)^. Já os escores mais baixos entre heterossexuais no domínio V podem ter relação com a baixa disponibilidade de informações e acesso aos serviços de saúde, pois como não pertencem às populações vulneráveis, não são o foco das ações preventivas do HIV^(38)^. Quanto a ter companheiro(a) e filhos, como o domínio V também aborda a segurança com a vida diária, recursos financeiros e lazer, destacam-se as questões socioeconômicas^(29)^, pois o aporte financeiro contribui para adesão à TARV, alimentação saudável e acesso à saúde^(39)^.

No domínio VI (espiritualidade), aqueles que viviam com três ou mais pessoas no domicílio tiveram escores inferiores. Como esse domínio aborda o medo do futuro, esses escores podem ocorrer pelas preocupações com o núcleo familiar, pois pessoas com HIV com filhos têm medo e estresse^(35)^. Não aceitar o diagnóstico teve relação com menores escores de QV; sobre isso, os estudos mostram que o amparo religioso contribui no enfrentamento da infecção pelo HIV^(40)^, influenciando positivamente a QV^(30)^. Não aceitar a sorologia anti-HIV positiva teve associação a menores escores em todos os domínios do WHOQOL-HIV-Bref. A aceitação do diagnóstico é importante para adesão à TARV e relaciona-se à QV satisfatória^(41)^, enquanto a negação pode acompanhar reações psicossociais negativas^(2)^. As pessoas com HIV que sofriam estigma tiveram escores inferiores em quase todos os domínios da QV, exceto no VI (espiritualidade). Os eventos psicológicos associados ao HIV podem ocorrer pelo estigma, que influencia negativamente a QV^(13,18)^, ressaltando-se a importância do suporte social^(17)^.

No domínio I, as pessoas com HIV com menor contagem de linfócitos T CD4+, carga viral detectável e insatisfeitas com o acompanhamento em saúde tiveram escores inferiores, o que mostra importância do controle imunológico com a TARV^(3,4)^. No domínio II, a menor satisfação com o acompanhamento em saúde teve relação com escores inferiores. Por abordar aspectos psicológicos, como satisfação pessoal e frequência de sentimentos negativos, observa-se a ocorrência de eventos depressivos em pessoas com HIV^(42)^, que podem aumentar os comportamentos de risco^(43)^. Quanto à insatisfação com os serviços de saúde, descentralizar o cuidado aumenta a satisfação dos usuários e melhora a adesão à TARV^(44)^.

O domínio III teve escores inferiores para os que não aceitavam o diagnóstico e sofriam estigma. Esse domínio avalia a necessidade de tratamento percebida, mostrando que devem-se abordar a não aceitação da doença e o atraso no tratamento, pois a adesão à TARV é afetada pelo estigma^(39)^, e aqueles que sofrem estigma têm mais problemas no mercado de trabalho^(32,33)^. Nos domínios IV e V, as pessoas com HIV com carga viral detectável e passagem no sistema prisional tiveram escores inferiores de QV, inferindo-se que o estigma e o histórico penitenciário dificultam o acesso aos cuidados de saúde^(45)^. Há risco aumentado de infecção pelo HIV em pessoas com antecedentes prisionais, pois o acesso à TARV e às medidas preventivas é um desafio nessas instituições^(46)^.

Por fim, esses dados mostram a importância da assistência integral às pessoas com HIV. O acompanhamento dos exames laboratoriais e busca das metas de parâmetros de carga viral e contagem de linfócitos T CD4+ são atividades essenciais desempenhadas pela equipe de saúde. Porém, avaliar as pessoas com HIV, de forma global, pode proporcionar a obtenção de informações capazes de orientar sobre as práticas de educação em saúde, para o bem-estar desses indivíduos e daqueles com os quais se relacionam.

### Limitações do estudo

Teve-se como limitação para realização do estudo a pandemia de COVID-19, a qual interferiu na coleta de dados, devido às restrições e alterações na dinâmica de trabalho nos serviços de saúde.

### Contribuições para as áreas da enfermagem, saúde, ou políticas públicas

A pesquisa forneceu dados para a compreensão de diferentes fatores que influenciam a QV das pessoas com HIV. Destacou-se a satisfação com o acompanhamento em saúde, que mostra a importância das consultas de seguimento pela equipe multidisciplinar, sobretudo o enfermeiro, que tem um papel primordial nas práticas de educação em saúde. Ademais, os resultados do estudo podem auxiliar no desenvolvimento de intervenções individuais e coletivas capazes de melhorar a QV, bem como orientar os participantes quanto ao autocuidado e às estratégias de prevenção combinadas do HIV.

## CONCLUSÕES

A maioria das pessoas com HIV teve QV satisfatória, sem diferença entre soroconcordantes e sorodiscordantes. Houve tendência de significância estatística no domínio III (nível de independência), em que aqueles com parceria sorodiscordante tiveram percepção mais intermediária da QV, comparados aos soroconcordantes. Ser do sexo feminino e não aceitar o diagnóstico teve associação à QV mais insatisfatória. Os desempregados, de licença/auxílio doença, com renda menor que um salário mínimo e que sofriam estigma tiveram escores inferiores de QV em quase todos os domínios, exceto espiritualidade/religião/crenças. Os heterossexuais, pessoas com mais de 40 anos, que viviam com companheiro(a), com menos de oito anos de estudo, que tinham filhos e mais de três pessoas no domicílio tiveram escores inferiores em vários domínios. As pessoas com HIV com contagem de linfócitos T CD4+ menor que 350 células/mm^3^, carga viral detectável, insatisfeitas com o acompanhamento em saúde e passagem no sistema prisional tiveram percepção mais inferior da QV. Esses dados mostram que apesar de a maioria dos participantes ter uma percepção superior da QV, ainda há aspectos que podem ser avaliados e trabalhados pela enfermagem e demais membros da equipe multiprofissional, visando ao bem-estar das pessoas com HIV e aos cuidados para não transmitir o vírus.

## Data Availability

Os dados da pesquisa estão disponíveis no corpo do artigo.
